# Number sense or working memory? The effect of two computer-based trainings on mathematical skills in elementary school

**DOI:** 10.5709/acp-0157-2

**Published:** 2014-06-09

**Authors:** Jörg-Tobias Kuhn, Heinz Holling

**Affiliations:** Institute of Psychology, University of Münster, Germany

**Keywords:** working memory training, number sense training, elementary school, arithmetics

## Abstract

Research on the improvement of elementary school mathematics has shown that computer-based training of number sense (e.g., processing magnitudes or locating numbers on the number line) can lead to substantial achievement gains in arithmetic skills. Recent studies, however, have highlighted that training domain-general cognitive abilities (e.g., working memory [WM]) may also improve mathematical achievement. This study addressed the question of whether a training of domain-specific number sense skills or domain-general WM abilities is more appropriate for improving mathematical abilities in elementary school. Fifty-nine children (*M*_age_ = 9 years, 32 girls and 27 boys) received either a computer-based, adaptive training of number sense (*n* = 20), WM skills (*n* = 19), or served as a control group (*n* = 20). The training duration was 20 min per day for 15 days. Before and after training, we measured mathematical ability using a curriculum-based math test, as well as spatial WM. For both training groups, we observed substantial increases in the math posttest compared to the control group (*d* = .54 for number sense skills training, *d* = .57 for WM training, respectively). Whereas the number sense group showed significant gains in arithmetical skills, the WM training group exhibited marginally significant gains in word problem solving. However, no training group showed significant posttest gains on the spatial WM task. Results indicate that a short training of either domain-specific or domain-general skills may result in reliable short-term training gains in math performance, although no stable training effects were found in the spatial WM task.

## Introduction

In modern society, mathematical ability is regarded as a key element for intellectual functioning. Deficits in mathematical knowledge may result in substantial impairments of factors like employment, educational achievement, or quality of life (Parsons & Bynner, 2005). For this reason, a number of interventions have been designed to foster the development of mathematical abilities. The design of these interventions has mainly been guided by research focusing on two related aspects: (a) basic numerical capacities, which have been shown to be precursor skills for elementary school mathematics, and (b) deficits in children with mathematical learning disabilities (MLD), because these deficits highlight potential developmental bottlenecks. Children with MLD suffer from deficits in basic numerical capacities like processing, comparing, and estimating numbers and numerosities. Consequently, they have difficulties in solving arithmetic problems or memorizing basic arithmetic facts. In MLD, these deficits are not attributable to low cognitive ability or the absence of adequate learning opportunities.

Several longitudinal studies have established the close relationship between basic numerical capacities and mathematical skills. For example, Aunola, Leskinen, Lerkkanen, and Nurmi (2004) showed in a longitudinal study that counting ability in kindergarten was a key predictor of math performance in Grade 2. With respect to older children, another study (Krajewski & Schneider, 2009) found that quantity to number-word linkage in preschool was a substantial predictor of math performance in Grade 4. The importance of quantity to number-word linkage has been reported in many studies, and it is a core component in models of mathematical development (e.g., Butterworth, 2010; von Aster & Shalev, 2007). Adding to these findings, a recent longitudinal study by LeFevre et al. (2010) has highlighted the importance of three important precursor skills in kindergarten for math performance in mid-elementary school: linguistic skills (e.g., phonological awareness), quantitative skills (e.g., dot counting), and spatial attention (e.g., spatial working memory). This result underscores the fact that in addition to basic numerical capacities, more general cognitive abilities are relevant in the development of mathematical skills.

MLD generally pertains to a deficit in number sense, which encompasses skills related to number processing, number relations, and number operations (Gersten, Jordan, & Flojo, 2005). Deficits in number sense manifest themselves in an impaired access or representation of numerosities (Butterworth, 2010). This “core deficit” affects all activities involving the processing of quantities, magnitudes, or numbers. The core deficit pertains to two very basic cognitive systems of number processing: the approximate number system (ANS) and the object tracking system (OTS). The ANS is utilized in processing large approximate numerosities (e.g., during estimation tasks), whereas the object tracking system is active when processing small exact numerosities (e.g., during enumeration or counting tasks; Feigenson, Dehaene, & Spelke, 2004). Both children with MLD and young children usually are relatively slow or imprecise in both symbolic (i.e., Arabic numbers) and nonsymbolic (e.g., clouds of dots) magnitude processing. For example, it has been shown that 10-year old children with MLD have the same acuity in discriminating numerosities of dots as 5-year old children without impairment (Piazza et al., 2010). Further, children with MLD are substantially slower in single-digit magnitude comparison tasks than an unimpaired control group (Landerl, Bevan, & Butterworth, 2004).

Another important deficit resulting from impaired number representation or access consists in a distorted mental number line. That is, children with MLD often overestimate the positions of small numbers on a number line and are generally less accurate in placing numbers on a number line than unimpaired children (Geary, Hoard, Nugent, & Byrd-Craven, 2008). It is assumed that a distorted mental number line, which lies at the heart of several theories of development of number processing (Dehaene, Piazza, Pinel, & Cohen, 2003), affects mathematical ability negatively (von Aster & Shalev, 2007). In line with these findings, Sasanguie, Göbel, Moll, Smets, and Reynvoet (2013) found that the precision of mapping numbers to a number line is predictive of mathematical ability in unimpaired elementary school children. In sum, deficits in number sense (e.g., estimation, number line, or counting tasks) have often been reported as a potential cause for low mathematical achievement or MLD, and longitudinal studies highlight the importance of basic numerical capacities for the development of mathematical abilities. Consequently, a training of these basic skills can be presumed to improve mathematical ability.

Whereas basic numerical capacities can be conceptualized as domain-specific predictors of mathematical skills, there is a growing body of research underscoring the importance of working memory (WM) as an important domain-general predictor (Geary, 2003). WM is usually conceived as a general cognitive ability which enables a person to hold information in mind while simultaneously performing other mental processes. The WM system is generally regarded as consisting of different subsystems (Baddeley, 1986). Two representational systems are concerned with storing information, one for verbal and language-related content (phonological loop) and another for visual information (visuospatial sketch pad). In contrast, the central executive represents the core system of WM. It is responsible for the attention-driven control of information (e.g., inhibiting irrelevant information, shifting attention, updating information). A large body of research has revealed that WM plays a key role in the development of arithmetic abilities (e.g., Friso-van den Bos, van der Ven, Kroesbergen, & van Luit, in press; von Aster & Shalev, 2007). Further, children with MLD often show a substantial deficit in some or all subsystems of WM (Raghubar, Barnes, & Hecht, 2010), although spatial WM seems to play a key role (Rotzer et al., 2009). Recently, Metcalfe, Ashkenazi, Rosenberg-Lee, and Menon (2013) related brain activity during arithmetic problem solving to components of WM. The authors found that visuospatial WM was the best predictor for arithmetic problem solving, followed by the central executive, and that activation differences in frontoparietal neuronal circuits while solving arithmetic problems were related to visuospatial WM capacity. In contrast, the phonological loop was the weakest predictor of arithmetic problem solving. This result is in line with the work of Dumontheil and Klingberg (2012), who found that differences in brain activity while working on a spatial WM task were predictive of math performance 2 years later. Further, there is a substantial body of research highlighting the importance of the central executive in math achievement (e.g., Bull & Scerif, 2001; Passolunghi & Pazzaglia, 2004; van der Ven, Kroesbergen, Boom, & Leseman, 2012). For example, van der Ven et al. (2012) could show in a longitudinal study with elementary school children that updating, a key component of the central executive, was highly predictive of math achievement, in contrast to inhibition or shifting. The meta-analysis by Friso-van den Bos et al. (in press) found that of all WM components, verbal up-dating correlated most strongly with math performance. The mentioned results generally suggest that spatial WM tasks as well as tasks tapping the central executive are promising candidates for an intervention to improve math performance.

Several computer-based interventions focusing on basic numerical abilities have been developed and empirically tested (Kroeger, Brown, & O’Brien, 2012; Slavin & Lake, 2008). For example, the number race program focuses on two key aspects: a deficit in nonsymbolic representation of number and the connection between symbolic and nonsymbolic quantities (Wilson, Dehaene, et al., 2006). The number race improved reaction times in dot enumeration and resulted in a substantially reduced number of subtraction errors. However, the program was not evaluated in comparison to an untrained control group. Another computer-based training, focusing on the mental number line, resulted in more linear number line representations and higher neuronal activation in areas relevant for number processing after training (Kucian et al., 2011). Further, a 6-week training of first graders, focusing on either approximate or exact magnitude representation, resulted in a substantial posttest gain in arithmetic (*d* = .40; Obersteiner, Reiss, & Ufer, 2013). Finally, a curriculum-based computer training of mathematical skills, conducted once a week for 10 weeks, resulted in a posttraining gain of *d* = .59 in first-graders and *d* = .62 in second-graders, respectively (Lenhard, Lenhard, Schug, & Kowalski, 2011). Computer-based trainings of basic numerical capacities and/or math skills therefore appear to result in reliable, small to medium short-term effect sizes.

A recent meta-analysis showed that WM training did generally not result in substantially improved arithmetic abilities or substantial transfer in general (Melby-Lervĺg & Hulme, 2013). However, some recent results that were not taken into account in this meta-analysis underline immediate or delayed positive effects of WM training on arithmetic abilities in unselected samples. For example, Holmes and Gathercole (in press) used the CogMed WM training (Klingberg, Forssberg, & Westerberg, 2002) to investigate the effects of WM training on scholastic achievement. The program contains visuo-spatial and verbal working memory tasks, and children have to complete a fixed number of trials (approx. 100) each day. After 20 training sessions, substantial gains in mathematics and reading were obtained (see also Dahlin, 2013). Witt (2011) conducted a 6-week WM training with elementary school students, which resulted in a substantial gain (*d* = .69) on a mathematics posttest (cf. Holmes et al., 2010). Henry, Messer, and Nash (in press) reported substantial near-transfer gains in WM tasks after a 6-week WM intervention, but they did not find any far-transfer short-term or long-term gains in mathematics. In contrast to these results obtained in a standard school population, a training study with low-working memory children (Dunning, Holmes, & Gathercole, 2013) resulted in posttest gains in WM tasks, but scholastic achievement was unaffected. Hence, up to now, no clear statements on the effectiveness of WM training on math performance can be made.

The goal of this study was to compare two computer-based trainings, focusing on basic numerical capacities (number sense) or WM components thought to underlie mathematical ability. For this purpose, we contrasted a domain-specific training of number sense, a domain-general WM training, and an untrained control group. We expected a training gain in arithmetic and number-related skills in the number sense training group, as in prior studies showing largely positive training effects. In contrast, we expected training gains for the WM training group only in mathematical areas with high WM demand (e.g., word problems; Lee, Ng, & Ng, 2009). Further, we did not expect training gains in a WM measure in the number sense training group, but in the WM training group.

## Subjects and methods

### Participants

Fifty-nine elementary school children from Grades 3-4 (*M*_age_ = 9, *SD* = 0.7, 32 girls and 27 boys) participated in the study (number sense training: *n* = 20, WM training: *n* = 19, control group: *n* = 20) at the beginning of the school year (September). Children came from four different classes in a German elementary school and were randomly allocated to groups. All children were native German speakers. Training duration was 20 min per day for 15 days (3 school weeks). The control group received regular lessons during the training slots. Parental consent was obtained for all children prior to the study.

### Materials and procedure

Before and after training, children’s math ability and spatial WM capacity were assessed. For this purpose, we used a group-administered, standardized math test (DEMAT; Krajewski, Liehm, & Schneider, 2004; Roick, Gölitz, & Hasselhorn, 2004) as well as a computer-based WM task suitable for children, the spatial WM task (Vock & Holling, 2008). The DEMAT consists of 9-10 subtests, depending on grade level, which cover core aspects of the mathematics curriculum in elementary school (e.g., basic arithmetics, word problems, and geometry). The DEMAT reliability is high (α = .83 to .93). In the spatial WM task used, children had to memorize one to four visual matrix patterns in a 3 × 3 matrix that were shown subsequently on screen for 3 s each. The patterns shown, however, had to be remembered in a rotated fashion, either rotated 90° clockwise or 90° counterclockwise. The spatial WM task was administered without time limits and had a satisfying reliability (α = .80 of the visuospatial scale, as reported in Vock & Holling, 2008). We chose the spatial WM task because, similar to at least two of the WM training tasks, it both comprised visuospatial and executive aspects, and therefore could be regarded as a measure of near transfer. Finally, we assessed general intelligence before the training, using the CFT 20-R (Weiß, 2006). This test focuses on fluid intelligence and consists of four subtests: series completion, classification, matrices, and topologies. The CFT 20-R has a reliability of .92 and takes about 20 min to administer. Informed consent was obtained from all parents prior to the study.

The number sense training consisted of two number-line tasks and a magnitude comparison task. The goal of the first number-line task was to locate a geometric form containing a single number (e.g., 15), a structured number of squares (shown in rows of 10), or a simple calculation (e.g., 14 + 3) on a number line shown at the bottom of the screen (cf. Kucian et al., 2011). The number line ranged from 0 to 20 or from 0 to 100. Initially, children were shown mainly single numbers and squares, whereas the number of simple calculations became larger in more difficult levels. Also, whereas children could take 15 s for each trial initially, they could work only 7 s per trial in the highest difficulty level. The second number-line task was similar to the first. Here, three cards containing numbers, structured squares (in rows of 10), or calculations were presented on the screen. All cards had to be drawn to their correct positions on the number line shown below (range 0-20 or 0-100, respectively). Initially, children were mainly presented numbers or squares, whereas higher difficulty levels introduced simple calculations on the cards used. At start, 20 s were given for each trial, whereas in later difficulty levels, only 10 s were provided. The main goal of this task was, in addition to locating single magnitudes on the number line, to foster an understanding of relations of magnitudes or numbers, which is regarded as an important step in developing mathematical skills (Krajewski & Schneider, 2009). The third number sense task implied a magnitude comparison (Wilson, Revkin, Cohen, Cohen, & Dehaene, 2006), in which the children were supposed to select (out of two or out of four) the card(s) with the overall larger magnitude, where magnitudes were shown as jittered squares, numbers, or calculations, respectively. Whereas nonsymbolic comparisons were mainly used in lower difficulty levels, number comparisons and simple sum comparisons were shown more frequently in higher difficulty levels. Children initially had 15 s per trial, later only 10 s were given. The main goal of this training task was to enhance quantity to number linkage in combination with calculation skills. Magnitudes in all three tasks ranged from 1 to 99. All of the number sense training tasks, therefore, focused on basic numerical capacities and arithmetic skills required in mathematics in elementary school.

The used WM training tasks mainly focused on spatial WM. The first WM training task was a spatial n-back (updating) task, in which the child was supposed to indicate whether a stimulus appearing on screen (a bolt of lightning in a cloud) was shown in the same cloud (out of six) as n steps before (Jaeggi, Buschkuehl, Jonides, & Shah, 2011). The flash of lighting was shown for 1 s, and the child had to answer within 5 s. This WM training task was supposed to enhance spatial updating, which has been shown to be of high importance in mathematics (van der Ven et al., 2012). The second WM training task was a Corsi block task in which a sequence of stars appeared in the cells of a 4 × 4 array, and the child should correctly repeat the sequence using the mouse (Passolunghi & Cornoldi, 2008). Each star was shown for 1 s, with an interstimulus interval of 1.5 s. In half of the trials, the sequence had to be memorized in the presented order, and in the other half of trials, it had to be memorized in reverse order. Hence, we assumed that this task tapped both the visuospatial sketchpad and the central executive. The third WM training task was a variant of the letter span task, in which a sequence of auditorily presented letters or numbers, each occurring in 50% of the trials, had to be memorized, of which a randomly-selected one had to be retrieved (Klingberg et al., 2002). Between letters or numbers, an interstimulus interval of 1 s was provided. Of all WM tasks, only the letter span task had numerical content, but no arithmetic was required in solving the tasks. The letter span task, even though it contains verbal content, can be assumed to tap spatial attention due to its requirement to memorize stimuli in a specific order (van Dijck, Abrahamse, Majerus, & Fias, 2013). However, spatial attention and spatial WM have been shown to be closely related (e.g., Awh & Jonides, 2001). We therefore assumed that this training task, although it was based on verbal content, also tapped the central executive and spatial attention in addition to the phonological loop.

**Figure 1.**
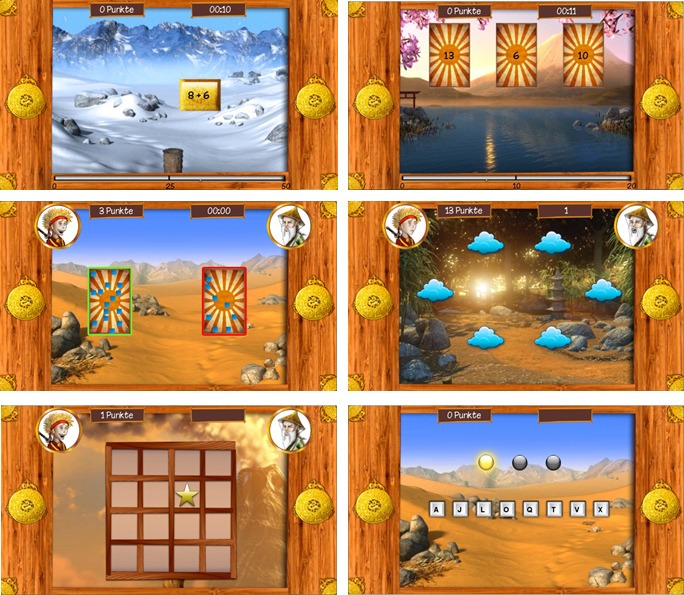
Screenshots of training tasks used. Upper panel: number line Tasks 1 and 2. Middle panel: numerosity comparison and *n*-back task. Lower panel: Corsi block task and letter span task.

Training difficulty was adapted individually by keeping accuracy levels between 70% and 80% per item block for each task in both the number sense and WM training. Figure 1 visualizes the training tasks used in this study. We used a balanced training design such that each child was presented two different training tasks a day, with each task occurring equally often across the entire training period. Two training tasks per day were used, with a training duration of 10 min per task. All tasks were explained auditorily (each child used headphones) to minimize reading load. After each trial, visual and auditory feedback was given.

In order to motivate children, a background story was constructed. Children were supposed to free Talasia, a fantasy kingdom that had been subdued by an evil dragon. At the beginning of the game, each child chose an avatar, a boy or a girl. With each finished training session, the child moved one step closer towards the dragon, as shown on a map. At the final training day, the child competed with the dragon in solving training tasks.

### Statistical analyses

In order to compare groups, we used ANOVAs as well as simple contrasts, comparing each single training group to the control group. We used posttest gains in the DEMAT total score, posttest gains in each scale score (arithmetic skills, word problems, geometry) as well as raw score gains in the WM tasks as the dependent variables to model training gains in math ability and spatial WM, respectively. Posttest gains were computed by subtracting pretest scores from posttest scores. The advantage of doing so lies in the fact that the between-subject *F*-value is not underestimated in this case (Huck & McLean, 1975). Although the groups did not differ significantly on any of the variables of interest at pretest, we used pretest IQ as well as the pretest *T*-scores of DEMAT, along with raw scores in spatial WM as covariates in subsequent ANCOVAs, using posttest gains as dependent variables. We did so to compensate for differences in pretest variables. Age was used as a co-variate only for the WM raw score gains, because the DEMAT *T*-scores were already age-adjusted.

We generally expected that both training groups would benefit from their respective training, and hence, we expected that both the number sense and WM training group would show larger posttest gains in math ability than the control group. However, we expected more stable gains in the number sense group. Also, a training gain in the spatial WM task was expected for the WM group because this training variant focused on spatial WM. We did not expect the number sense training to differ from the control group in WM gains.

In order to test these hypotheses, we computed planned contrasts, contrasting both the number sense training group and the WM group with the control group, respectively. Because the planned comparisons were dependent, we corrected the respective *p*-values using the Bonferroni-Holm method (i.e., for two comparisons, *p* = .025 for the first comparison, *p* = .05 for the second comparison; Holm, 1979). In order to assess the magnitude of training gains, we computed two variants of Cohen’s *d*. The first, more conservative between-groups variant (*d*_between_) compared gains in the training groups to those in the control group (Morris, 2008), whereas the second variant compared posttest to pretest scores within groups (*d*_within_). Both effect size measures were based on (pooled) pretest standard deviations.

## Results

Descriptive statistics of all study measures by group and sample are shown in Table 1. For all samples, we compared group means with multiple ANOVAs using the Bonferroni-Holm method for correcting alpha inflation mentioned above. This resulted in non-significant mean differences for all mean comparisons. The distribution of boys and girls was comparable across groups, χ^2^(2) = 3.27, *p* = .195.

**Table 1.** Means of Study Measures by Group

Study measure	CG(*n* = 20)	WM(*n* = 19)	NS(*n* = 20)	*F* (*df*)	*p*
DEMAT^a^, pretest	53,10 (8,28)	51,63 (7,45)	51,35 (8,66)	0,264 (2, 56)	.769
DEMAT^a^, posttest	52,00 (8,55)	55,05 (10,02)	54,60 (7,59)	0,699 (2, 56)	.501
Spatial WM, pretest	2,55 (1,90)	2,11 (1,97)	2,40 (2,01)	0,258 (2, 56)	.774
Spatial WM, posttest	3,55 (1,64)	3,11 (1,59)	3,10 (2,36)	0,963 (2, 36,655)^c^	.650
IQ^b^, pretest	110,25 (17,82)	105,32 (14,35)	106,40 (10,98)	0,616 (2, 56)	.543
Age (in months)	110,40 (9,12)	105,74 (8,54)	106,00 (7,36)	1,940 (2, 56)	.153

*Note*. CG = control group, NS = number sense training group, WM = working memory training group. Standard deviations in parentheses. ^a^ *T*-values (*M* = 50, *SD* = 10). ^b^ IQ values (*M* = 100, *SD* = 15), based on CFT 20-R. ^c^ Due to heterogeneous variances between groups, the Welch procedure was used.

**Figure 2.**
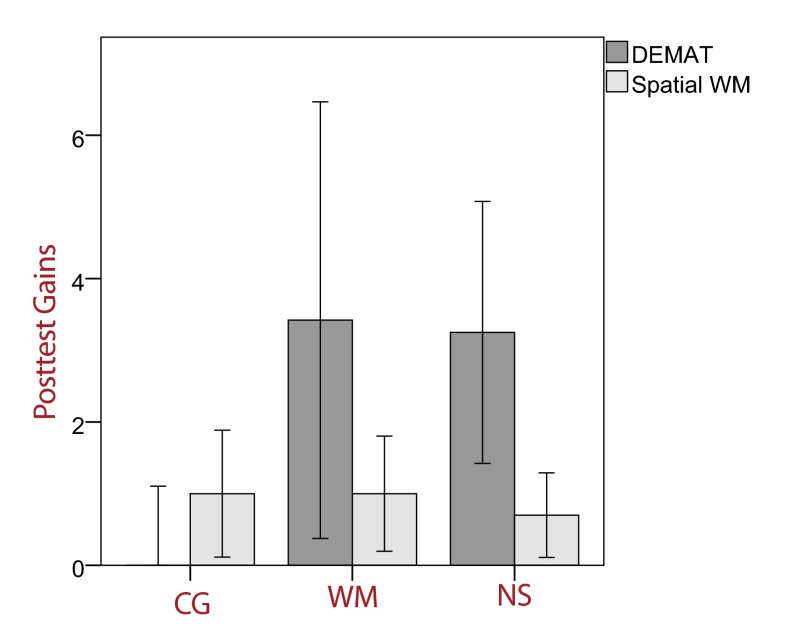
Posttest gains DEMAT (*T*-scores) and spatial working memory (raw scores) with 95% confidence intervals. CG = control group, WM = working memory training group, NS = number sense training group.

Training gains on all tasks are visualized in Figure 2. A one-way ANOVA revealed substantial group differences in training gains in the DEMAT, *F*(2, 56) = 5.93, η_p_^2^ = .15, *p* < .01. Planned contrasts showed that both the number sense group and the WM training group exhibited significantly higher training gains than the control group, *t*(38) = 2.71, *p* < .01, and *t*(37) = 2.80, *p* < .01, respectively. The gain score of the control group did not substantially differ from zero, *t*(19) = -1.04, *p* = .31. This pattern of results remained virtually unchanged in the case that IQ, DEMAT pretest score, and spatial WM pretest score were used as covariates, *F*(2, 53) = 4.93, η_p_^2^ = .16, *p* < .05.Planned contrasts produced similar results to those obtained before. Effect sizes are shown in Table 2. As can be seen, a reliable, small training gain in DEMAT scores was found for both training groups, whereas the control group did not show any significant change.

**Table 2.** Effect Sizes for Posttest Training Gains

	*d*_between_		*d*_within_		
	WM-CG	NS-CG	CG	WM	NS
DEMAT	.57*	.54*	-.13	.46*	.38*
Spatial WM	.00	-.15^a^	.53*	.51*	.35*

*Note*. CG = control group, WM = working memory training group, NS = number sense training group. a Control group had higher gains than training group. *p < .05.

Next, we investigated gains in spatial WM. We did not find any reliable group differences in posttest gains, *F*(2, 56) = 0.23, *p* = .799. However, post-hoc *t*-test revealed that each group substantially improved in the spatial WM task: *t*(19) = 2.36, *p* < .05 (the control group); *t*(18) = 2.62, *p* < .05 (the WM training group); *t*(19) = 2.48, *p* < .05 (the number sense group). Results remained unchanged when taking pretest scores, age, and IQ into account. However, all training groups tended to obtain higher posttest gains, with significant within-group gains across all groups (see Table 2).

**Figure 3.**
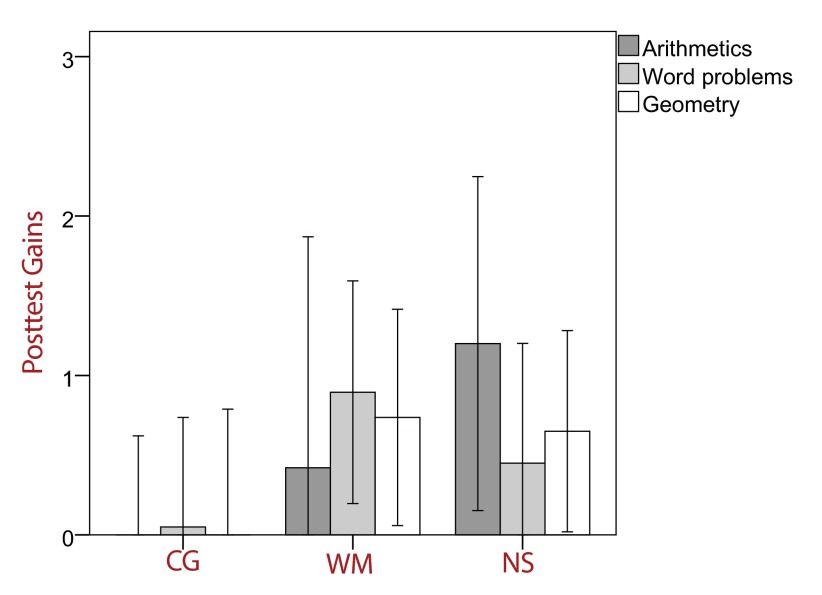
Posttest gains DEMAT subscales (*T*-scores) with 95% confidence intervals. CG = control group, WM = working memory training group, NS = number sense training group.

Finally, we analyzed whether training gains in the DEMAT were homogeneous across subscales (arithmetic skills, word problems, geometry). Figure 3 shows posttest gains on the DEMAT subscales by group. Planned contrasts revealed that in the arithmetics subscale, the number sense group significantly differed from the control group, *t*(38) = 1.71, *p* < .05, whereas the WM group did not, *t*(37) =1.55, *p* = .25. In contrast, planned comparisons pertaining to word problems revealed the opposite pattern, with a marginally significant contrast for the WM group, *t*(37) = 1.84, *p* = .07, and a non-significant contrast for the number sense group, *t*(38) = 0.79, *p* = .44. No significant group differences were found for geometry.

## Discussion

The goal of the current study was to investigate the effects of a computer-based, adaptive training of either number sense or WM on math ability and spatial WM capacity, respectively. Our findings showed that both training variants led to substantial but small gains in math ability, although no stable evidence was found for gains in spatial WM capacity. Overall, both training variants appeared beneficial for improving DEMAT posttest scores. The results are in line with more recent studies, showing that either training of number sense or WM training may enhance math ability (Holmes & Gathercole, in press; Kucian et al., 2011; Witt, 2011). However, training time was comparatively short in this study, with a total training duration of 5 hr only, thus resulting in small but reliable gains in posttest math scores.

An interesting finding emerged when analyzing the subscales of the DEMAT. We found that whereas the number sense training resulted in improved arithmetic skills, the WM produced marginally significant gains in word problem solving. This is not an unexpected result, as word problem solving relies heavily on WM (Lee et al., 2009), whereas simple calculation, at least in a sample of unimpaired children, is much less dependent of WM. This result suggests interesting implications for interventions. For example, whereas deficits in arithmetic skills may be addressed in the context of number sense training, problems with word problem solving may require some kind of WM training. Interestingly, a recent study (Loosli, Buschkuehl, Perrig, & Jaeggi, 2012) found that a short WM training with a complex span task substantially improved reading skills in typically developing elementary school children. One of the reasons for the improvement of our WM training group in solving word problems may therefore have been an improvement in reading skills, as both WM and reading are closely related to attention control (Hasher, Lustig, & Zacks, 2008). Unfortunately, we did not investigate reading skills in this study, such that a more detailed causal account of the training gains cannot be given, necessitating further research.

We did not find reliable gains in the spatial WM measure used. There are several possible explanations for this pattern of findings. First, the number sense training comprised tasks that shared some key aspects with the DEMAT (e.g., simple calculations). However, the WM training tasks differed more strongly from the WM tasks used at pretest and posttest. It may be argued that the spatial WM task requiring mental rotation tapped a different component of the visuospatial sketchpad, the visual cache, more heavily than the WM training tasks. The latter require memorizing or updating spatial information, respectively, thus focusing on dynamic aspects that are more closely related to the inner scribe component (Logie, 2011). Additionally, it should be noted that the spatial WM task we used at pretest and posttest was relatively difficult, with less than 25% of items solved correctly on average. Hence, the sensitivity of this task may have been too low for measuring training gains.

The study has several limitations. First, the training variants presented here were, at least in the lower training levels which comprised easier tasks, primarily designed for children with MLD by focusing on basic numerical capacities. Thus, training of math ability was restricted to more fundamental aspects of number processing and simple calculations. However, we used an unselected sample here, in order to provide a broader picture of number sense training gains in a sample of elementary school children with varying levels of arithmetic ability. Nevertheless, the training gains observed were substantial, highlighting the fact that training basic numerical capacities can be beneficial even in normally developing children. Second, we did not use an active control group here. Instead, children in the control group received regular lessons in school. Hence, we cannot rule out motivational losses or other factors potentially confounding treatment effects. And third, training duration was relatively short, and no follow-up measurement was conducted. Hence, only statements of short-term training effects can be made.

To conclude, we found that a relatively brief training of number sense skills or WM capacity may result in small but reliable posttest gains in a curriculum-based math test. In contrast, WM capacity appeared more difficult to modify. More research is needed to corroborate and clarify causal mechanisms of this pattern of results, especially by taking longer training durations, additional transfer tasks, and samples of children with MLD into account.
